# CCL21/CCR7 axis regulating juvenile cartilage repair can enhance cartilage healing in adults

**DOI:** 10.1038/s41598-019-41621-3

**Published:** 2019-03-26

**Authors:** Zenta Joutoku, Tomohiro Onodera, Masatake Matsuoka, Kentaro Homan, Daisuke Momma, Rikiya Baba, Kazutoshi Hontani, Masanari Hamasaki, Shinji Matsubara, Ryosuke Hishimura, Norimasa Iwasaki

**Affiliations:** 1 Department of Orthopaedic Surgery, Faculty of Medicine and Graduate School of Medicine, Hokkaido University, Sapporo, Japan; 2 Global Station for Soft Matter, Global Institution for Collaborative Research and Education (GSS, GI-CoRE), Hokkaido University, Sapporo, Japan

## Abstract

Juvenile tissue healing is capable of extensive scarless healing that is distinct from the scar-forming process of the adult healing response. Although many growth factors can be found in the juvenile healing process, the molecular mechanisms of juvenile tissue healing are poorly understood. Here we show that juvenile mice deficient in the chemokine receptor CCR7 exhibit diminished large-scale healing potential, whereas CCR7-depleted adult mice undergo normal scar-forming healing similar to wild type mice. In addition, the CCR7 ligand CCL21 was transiently expressed around damaged cartilage in juvenile mice, whereas it is rarely expressed in adults. Notably, exogenous CCL21 administration to adults decreased scar-forming healing and enhanced hyaline-cartilage repair in rabbit osteochondral defects. Our data indicate that the CCL21/CCR7 axis may play a role in the molecular control mechanism of juvenile cartilage repair, raising the possibility that agents modulating the production of CCL21 *in vivo* can improve the quality of cartilage repair in adults. Such a strategy may prevent post-traumatic arthritis by mimicking the self-repair in juvenile individuals.

## Introduction

Self-repair in juveniles is more extensive and scarless than in mature individuals^1^. Since articular cartilage in adults has a limited potential for repair because of the insufficient presence of stem cells, and its structural features, cartilage injury can lead to osteoarthritis, resulting in millions of patients experiencing pain around the world^2^. We have previously reported that cartilage tissues in adult mice showed low self-repair potential, whereas juvenile mice demonstrated hyaline-cartilage repair at the site of cartilage injury^3^. This suggests that clarifying the mechanisms of juvenile cartilage healing may improve the fate of adult cartilage injuries. However, the mechanistic differences in cartilage repair and chondrogenic potential with age are unknown.

To clarify the chondrogenic potential of bone marrow stromal cells and certain progenitor cells in juveniles, we focused on chemokines because they regulate the migration of stem cells and progenitor cells during tissue homeostasis^4,5^. In addition, the expression pattern of chemokines is known to change with age^6–8^. Chemokines are a family of small molecules of approximately 8–10 kDa in size, with more than 45 chemokines and 19 receptors identified to date. Chemokines are classified into four groups based on the number and spacing of their cysteine residues: CC, CXC, C, and CX_3_C^9,10^. We have previously clarified that CXCL12 enhances the repair process of osteochondral injuries through increased migration of host cells to the injury site^11^. Several studies have reported the involvement of chemokines in cartilage repair^12–14^, however, it remains unknown which chemokines are mainly involved in cartilage repair.

We hypothesized that the articular cartilage repair process in juvenile animals may be regulated by specific chemokines. To test this hypothesis, we performed a comprehensive screening of chemokines involved in cartilage repair. Our screening detected the CCL21/CCR7 axis as a candidate chemokine involved in self-repair. Subsequently, we generated osteochondral injury in CCR7 knockout (CCR7^−/−^) mice to analyse the articular cartilage repair potential. Finally, we evaluated cartilage repair by administration of CCL21 in a rabbit osteochondral defect model. In this study, juvenile CCR7-deficient mice exhibited a diminished healing potential, whereas adult CCR7-deficient mice showed normal scar-forming repair. In addition, CCL21 administration enhanced hyaline-cartilage repair in an adult rabbit model. Our findings demonstrate that the CCL21/CCR7 axis may constitute a part of the molecular control mechanism of juvenile cartilage repair, raising the possibility that agents modulating the production of CCL21 *in vivo* can improve the quality of cartilage repair in adults. This approach may have a great impact on cartilage repair therapy by mimicking self-repair in juveniles.

## Results

### CCL21/CCR7 axis was detected as a chemokine involved in self-repair in juveniles

**Figure 1:**
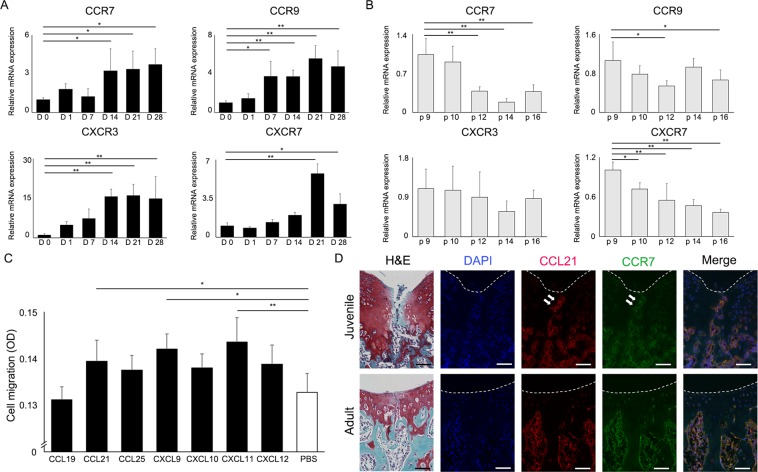
The CCL21/CCR7 axis was identified as a candidate target related to the cartilage repair mechanisms in juvenile animals. (**A**) Changes in the gene expression of chemokine receptors induced by chondrogenic differentiation. The mRNA expression patterns in cartilage differentiation (Day 0 to 28) were analysed (n = 3/group at each time point). All values are expressed as mean ± SD. *P < 0.05, **P < 0.01, significantly different compared to mRNA expression at D 0 (ANOVA followed by Tukey’s post hoc test). (**B**) Changes in the gene expression of chemokine receptors induced by long-term cultivation of MSCs. The mRNA expression patterns in long-term cultivation of MSCs in monolayer culture (passages 9 to 16) were analysed (n = 3/group at each time point). All values are expressed as mean ± SD. *P < 0.05, **P < 0.01, significantly different compared to mRNA expression of p 9 (ANOVA followed by Tukey’s post hoc test). (**C**) Cell migration assays were performed with the addition of the 7 chemokines corresponding to the receptors identified in the initial screening to the culture medium (n = 10/group). All values are expressed as mean ± SD. *P < 0.05, **P < 0.01, statistically significant (ANOVA followed by Tukey’s post hoc test). (**D**) CCL21 and CCR7 expression following osteochondral injury in WT mice (juvenile, upper row; adult, lower row). Representative images of immunohistochemistry for CCL21 (red) and CCR7 (green) in WT mice (juvenile, 3-week old; adult, 8-week old). CCL21, CCR7 were expressed at the injury sites of juvenile mice (white arrow). Scale bar: 50 µm.

We initially confirmed chondrogenic differentiation induced by micromass culture before profiling gene expression patterns of chemokine receptors. During chondrogenic differentiation from mesenchymal stem cells (MSCs), mRNA expression of chondrocyte-specific genes increased with time (Supplementary Fig. S1). The mRNA expression of the 4 chemokine receptors (CCR7, CCR9, CXCR3, and CXCR7) increased with cartilage differentiation, and decreased or did not increase with long-term cultivation of MSCs (Fig. 1A,B). Of the 7 chemokines corresponding to the receptors identified in the first screen, 3 chemokines (CCL21, CXCL9, and CXCL11) significantly enhanced MSCs migration (Fig. 1C). To clarify the mechanisms of osteochondral repair in juvenile mice, we analysed the expression patterns of CCL21 and CCR7 in juvenile and adult mice. Immunohistochemical analysis revealed that CCL21 was broadly expressed in the articular cartilage and bone marrow of juvenile and adult mice. In articular cartilage, juvenile mice showed high CCL21 expression compared to adult mice (Supplementary Fig. S2). CCL21 and CCR7 were increased at the injury site and broadly detected in the bone marrow of juvenile WT mice at 3 days postoperatively. In contrast, although CCL21 and CCR7 were detected in the bone marrow of adult WT mice, they were largely absent at the injury site (Fig. 1D). CCL21 positive cells accumulated at the injury site in juvenile mice, whereas almost no cells could be found in the damaged area in adult mice (Supplementary Fig. S3). Our comprehensive screening determined that the CCL21/CCR7 axis was a candidate target related to the self-repair mechanism in juvenile animals.

### Elimination of CCR7 in mice diminishes juvenile cartilage healing

We evaluated the growth phenotype of CCR7^−/−^ mice before evaluating the healing phenotype of the CCL21/CCR7 axis. Based on these results, we concluded that the CCR7^−/−^ mice developed and grew normally and could not be distinguished from their WT littermates (Supplementary Fig. S4).

**Figure 2:**
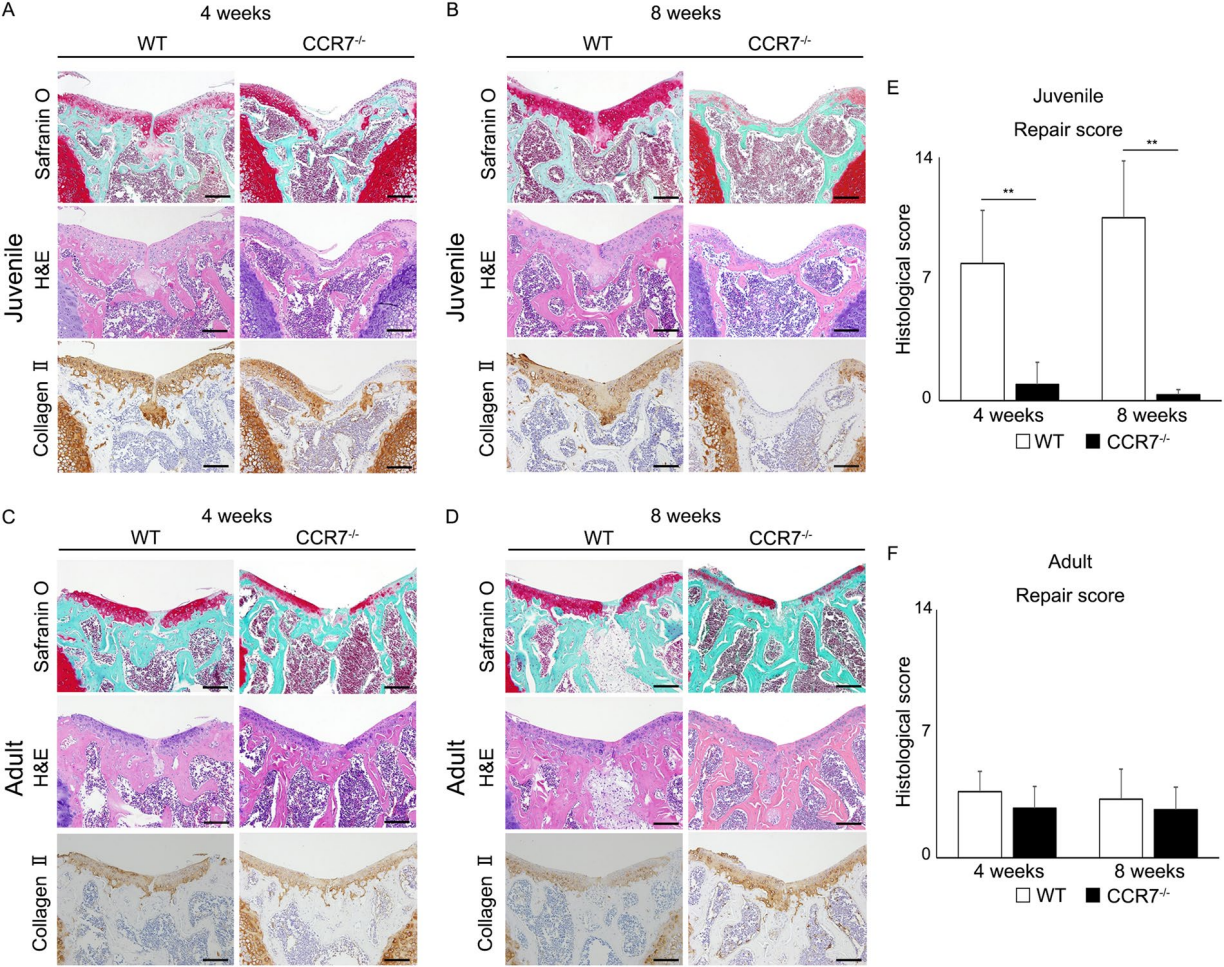
Elimination of CCR7 in comprehensive screening of juvenile mice diminishes enormous healing potential. (**A**,**B**) Representative images of the patellar groove surface in juvenile WT, CCR7^−/−^ mice with safranin O staining, H&E staining and immunostaining for type II collagen at 4 and 8 weeks postoperatively (n = 10/each group). Scale bar: 100 µm. (**C**,**D**) Representative images of the patellar groove surface in adult WT, CCR7^−/−^ mice with safranin O staining, H&E staining and immunostaining for type II collagen at 4 and 8 weeks postoperatively (n = 10/group). Scale bar: 100 µm. (**E**,**F**) Histological scores in juvenile and adult WT, CCR7^−/−^ mice at 4 and 8 weeks postoperatively (n = 10/group). Repair scoring range: 0–14. All values are expressed as mean ± SD (n = 10/group). **P < 0.01, statistically significant (Steel-Dwass test).

To confirm the relationship between the CCL21/CCR7 axis and juvenile cartilage repair, we analysed articular cartilage repair at 4 and 8 weeks postoperatively in WT and CCR7^−/−^ mice. Surprisingly, juvenile CCR7^−/−^ mice exhibited a dramatic loss of the characteristic cartilage healing potential at each time point, whereas juvenile WT exhibited self-repair with hyaline-like cartilage tissue. In juvenile WT mice, the injured areas were almost completely filled with reparative tissue poorly stained with safranin O at 4 weeks postoperatively. Although safranin O staining was slightly decreased around the injured area, the cartilage structure was mostly maintained without destruction (Fig. 2A. left panel). At 8 weeks postoperatively, the injured areas were completely filled with reparative tissue and the articular surface was smoother compared with that at 4 weeks postoperatively. Safranin O staining around the injured area was recovered and the reparative tissue seemed to integrate into the surrounding cartilage tissue (Fig. 2B, left panel). In juvenile CCR7^−/−^ mice, the injured areas were partially filled with subchondral bone covered by a thin fibrous layer poorly stained by safranin O and collagen II at 4 weeks postoperatively. In addition, cartilage destruction was observed around the injured area (Fig. 2A, right panel). At 8 weeks postoperatively, safranin O staining showed that cartilage destruction progressed over the entire patellar groove. The subchondral bone was widely exposed on the surface of the articular cartilage and was covered with a fibrous layer. Collagen II staining in the articular cartilage was widely diminished in the entire patellar groove (Fig. 2B, right panel). In juvenile mice, the repair score for the articular joint surface in CCR7^−/−^ mice was significantly lower than in the WT mice (Fig. 2E). Meanwhile, the femoral condyle after osteochondral injury showed slight degenerative changes. There was no significant difference between WT mice and CCR7^−/−^ mice (Supplementary Fig. S5). Conversely, the capacity for cartilage repair was almost identical between adult WT and CCR7^−/−^ mice. The injured area exhibited fibrous cartilage with faint safranin O staining, although type II collagen staining was observed. In adult WT and CCR7^−/−^ mice, the width of the injured area was wider than that of juvenile WT, and the area was poorly integrated with the surrounding cartilage tissue (Fig. 2C,D). In adult mice, there was no significant difference in repair score between CCR7^−/−^ and WT mice (Fig. 2F). These data revealed that the elimination of CCR7 caused a failure of the cartilage repair mechanism in juvenile mice.

### CCL21, CCR7 are expressed at the injury site during the early phase after osteochondral injury in juvenile mice

**Figure 3:**
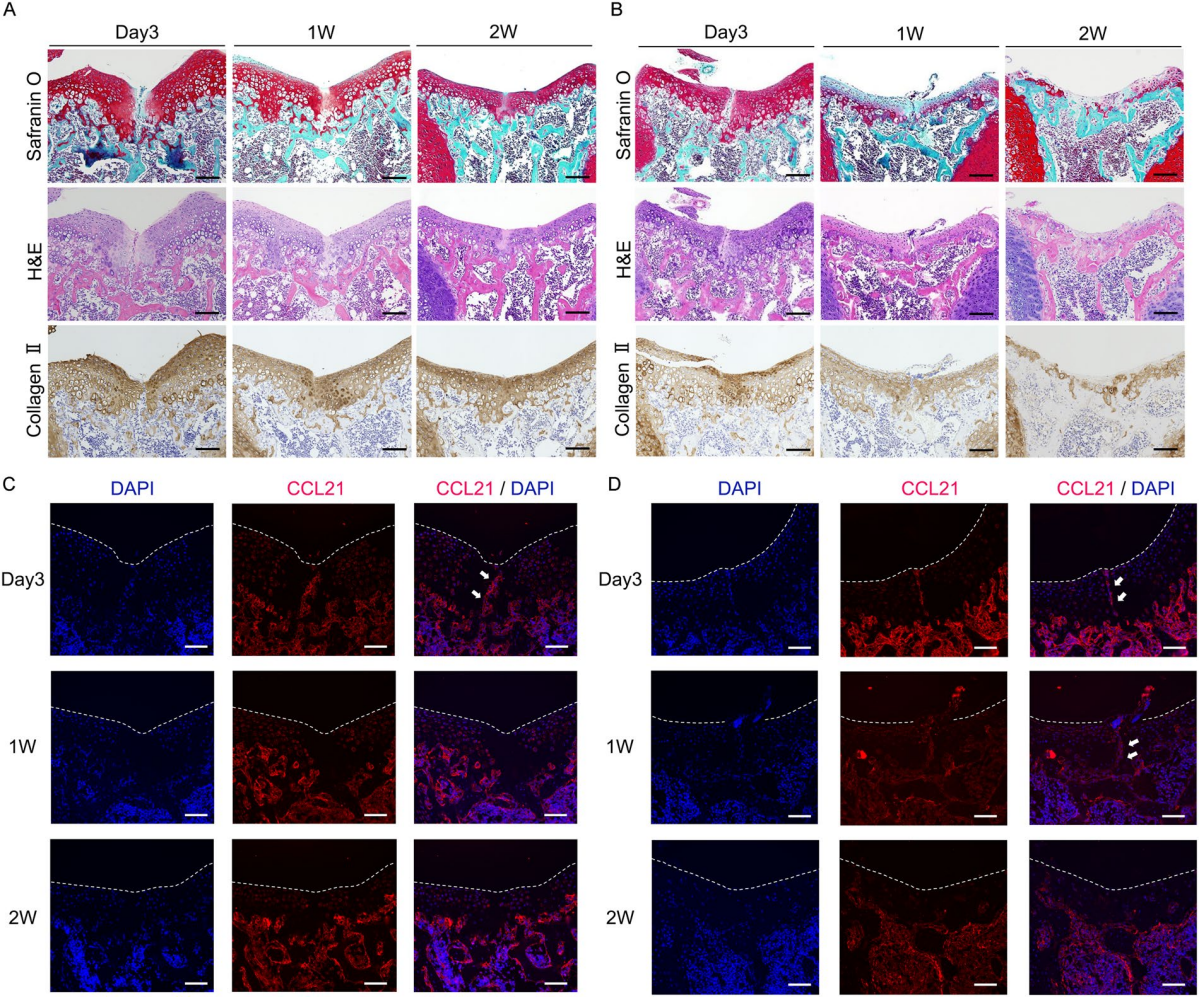
CCL21, CCR7 are expressed at injured sites of juvenile mice in the early phase of post injury. (**A**) Representative histology in juvenile WT mice with safranin O staining, H&E staining, immunostaining for type II collagen at 3 days, 1 week, and 2 weeks postoperatively. Scale bar: 100 µm. (**B**) Representative histology in juvenile CCR7^−/−^ mice with safranin O staining, H&E staining, immunostaining for type II collagen at 3 days, 1 week, and 2 weeks postoperatively. Scale bar: 100 µm. (**C**) Representative images for immunohistochemistry of CCL21 (red) in juvenile WT mice at 3 days, 1 week, and 2 weeks postoperatively. CCL21 was transiently expressed at the injury site in WT mice in the early phase of post injury (white arrow). Scale bar: 100 µm. (**D**) Representative images for immunohistochemistry of CCL21 (red) in juvenile CCR7^−/−^ mice at 3 days, 1 week, and 2 weeks postoperatively. CCL21 was transiently expressed at the injury site in CCR7^−/−^ mice in the early phase of post injury (white arrow). Scale bar: 100 µm.

At 3 days postoperatively in WT mice, safranin O staining was decreased around the injury area. At 2 weeks postoperatively, safranin O staining of the altered extracellular matrix gradually increased except at the centre of the injured area. Hematoxylin and eosin (H&E) staining revealed that the injured area was filled with scar tissue including high-density cells at 3 days postoperatively. The superficial layer of the joint surface exhibited irregularities and fibrillar discontinuities at 3 days postoperatively. After one week postoperatively, the joint surface became smooth (Fig. 3A). In CCR7^−/−^ mice, the injured area at 3 days postoperatively was similar to WT. However, the superficial layer of the injured area was covered by fibrous tissue that was faintly stained with safranin O at one week postoperatively. At 2 weeks postoperatively, cartilage around the damaged area was markedly degenerated without evidence of healing (Fig. 3B). In WT mice, the expression of collagen II was initially decreased around the injury area at day 3 and subsequently increased at 1 week (Fig. 3A lower panel). In CCR7^−/−^ mice, collagen II showed higher expression around the injury site at day 3 and subsequently decreased at 1 week (Fig. 3B lower panel).

These data suggest that cartilage destruction after osteochondral injury in CCR7^−/−^ mice occurred rapidly, around one week postoperatively. Immunohistochemical analysis revealed that CCL21 in WT mice was transiently increased at the injury site at 3 days and subsequently diminished at one week. In contrast, CCL21 in CCR7^−/−^ mice was continuously increased at the injured site from 3 days to one week postoperatively (Fig. 3C,D). Based on our results, cartilage destruction in CCR7^−/−^ mice was thought to occur rapidly around one week postoperatively, possibly due to the maintained expression of CCL21 in CCR7^−/−^. These data also suggest that the CCL21/CCR7 axis is associated with the cartilage repair process in juvenile mice.

### CCL21 significantly increases the migration of MSCs

**Figure 4:**
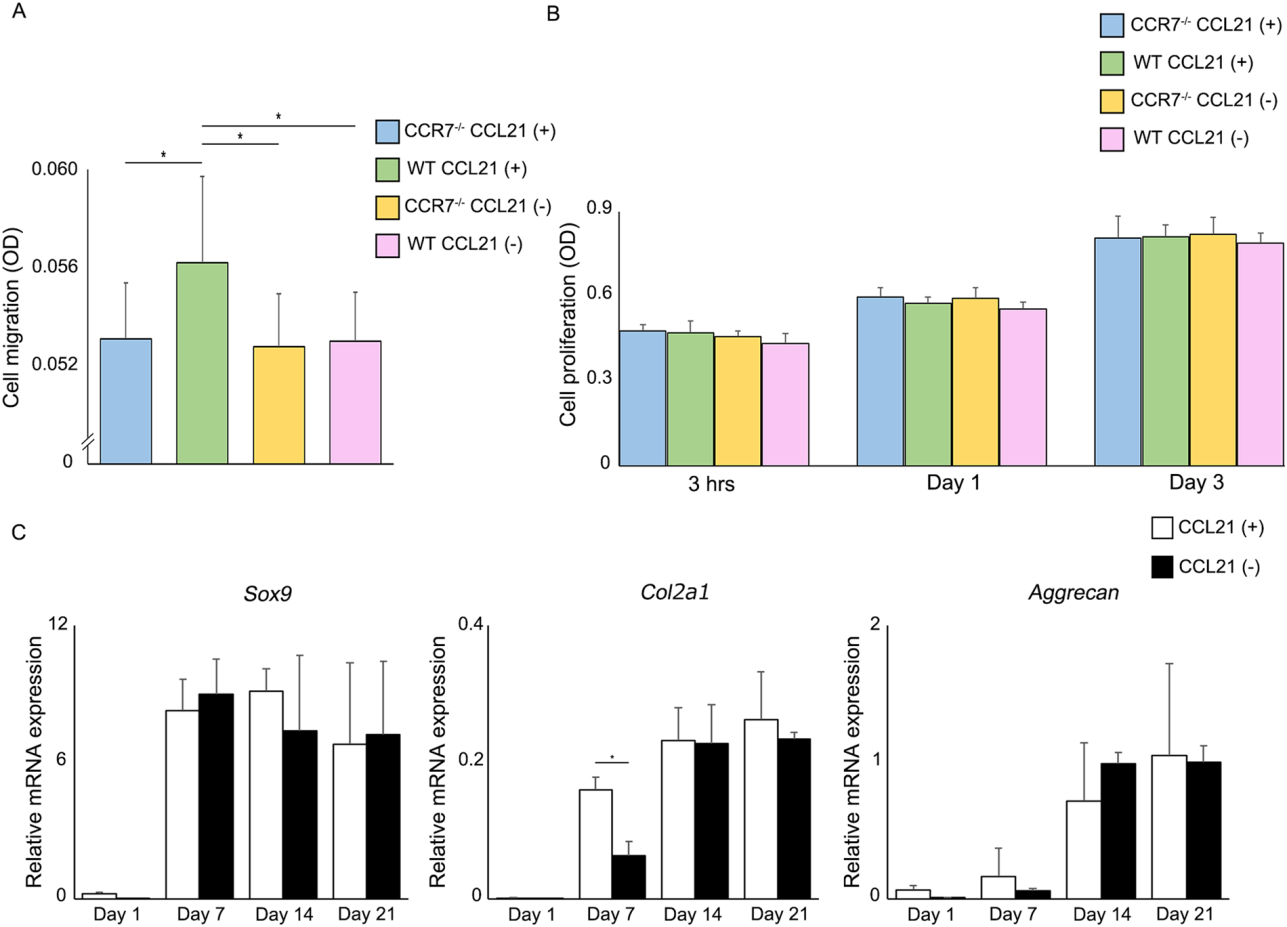
Association between elimination of CCR7 and behaviour of MSCs *in vitro*. (**A**) The effect of CCL21 (100 ng/ml) on cell migration (n = 10/group). All values are expressed as mean ± SD. *P < 0.05, statistically significant (ANOVA followed by Tukey’s post hoc test). (**B**) The effect of CCL21 (100 ng/ml) on cell proliferation (n = 10/group at each time point). All values are expressed as mean ± SD. (**C**) MSCs were differentiated in chondrogenic medium by micromass culture. qRT-PCR analysis of gene expression during the chondrogenic differentiation process (n = 3/each group at each time point). All values are expressed as mean ± SD. *P < 0.05, statistically significant (ANOVA followed by Tukey’s post hoc test).

*In vitro* evaluation using MSCs isolated from WT and CCR7^−/−^ mice revealed that CCL21 significantly increased MSC migration in WT, but not in CCR7^−/−^ mice (Fig. 4A). This result suggested that CCL21 utilizes the CCR7 receptor to regulate cell migration. The cell proliferation assay showed no significant difference at any time point between the experimental groups (Fig. 4B), indicating that the elimination of CCR7 does not affect cell proliferation. Additionally, CCL21 did not affect cell migration and cell proliferation in chondrocytes (Supplementary Fig. S6). Our results also revealed that exogenous CCL21 does not increase cell proliferation in WT and CCR7^−/−^ MSCs. Based on our results, we concluded that the CCL21/CCR7 axis is not involved in regulating cell proliferation. In a chondrogenic differentiation assay, exogenous CCL21 significantly increased the expression of *Col2a1* at 7 days after chondrogenic differentiation. However, there was no difference in *Col2a1* expression at days 14 and 21. In addition, the expression patterns of other cartilage differentiation markers, i.e., *Sox9* and *aggrecan*, were also identical between both groups (Fig. 4C). Based on our results, we concluded that the CCL21/CCR7 axis regulated the migration of MSCs whereas it was rarely associated with chondrogenic differentiation.

### Exogenous CCL21 administration enhances cartilage repair in rabbits

**Figure 5:**
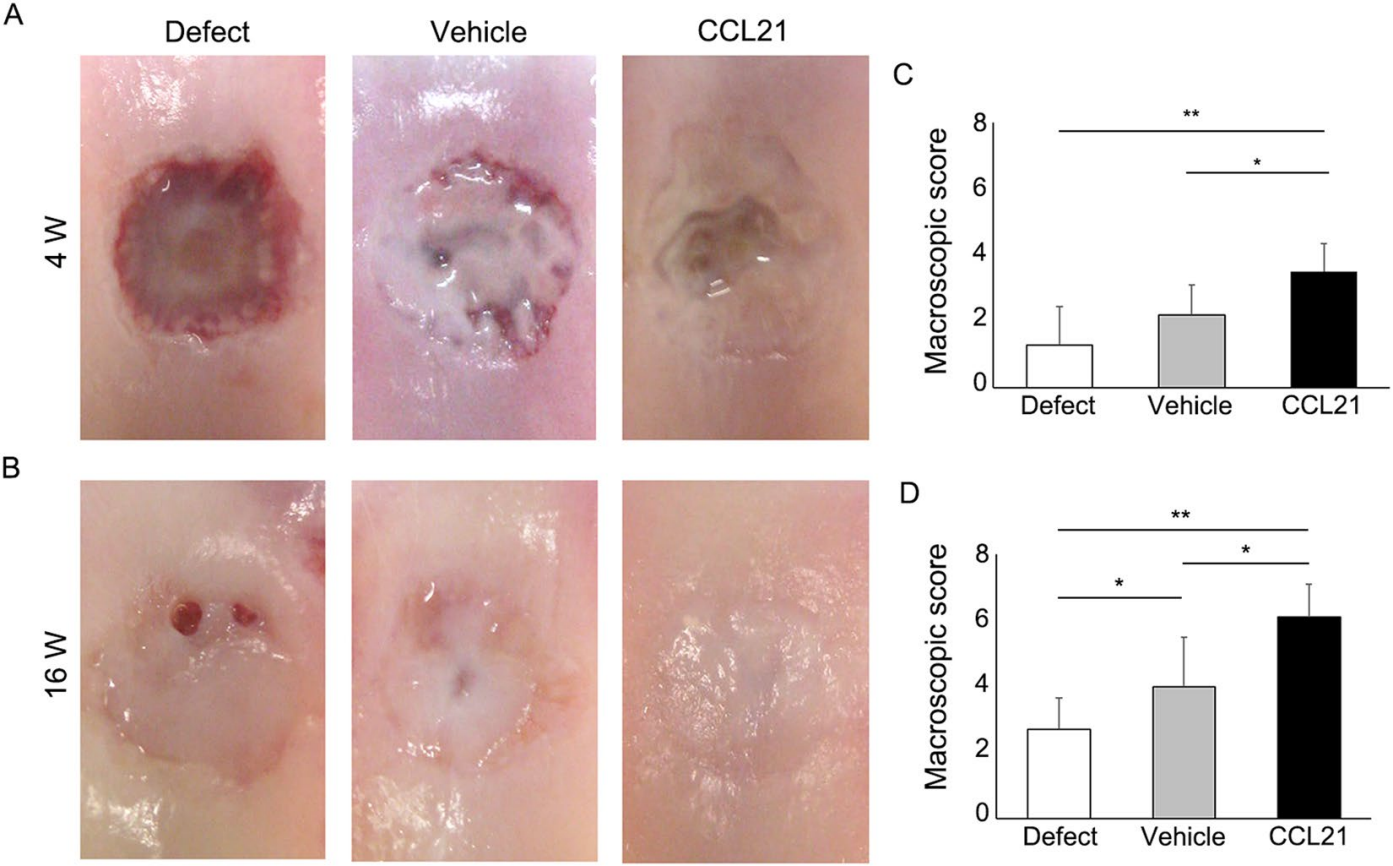
Exogenous CCL21 administration macroscopically enhances cartilage repair in rabbits. (**A**) Macroscopic morphology of the osteochondral defect at 4 weeks postoperatively (n = 10/group). (**B**) Macroscopic morphology of the osteochondral defect at 16 weeks postoperatively (n = 10/group). (**C**) Macroscopic scores at 4 weeks postoperatively (n = 10/group). (**D**) Macroscopic scores at 16 weeks postoperatively (n = 10/group). Repair scoring range: 0–8. All values are expressed as mean ± SD. *P < 0.05, **P < 0.01, statistically significant (Steel-Dwass test).

**Figure 6:**
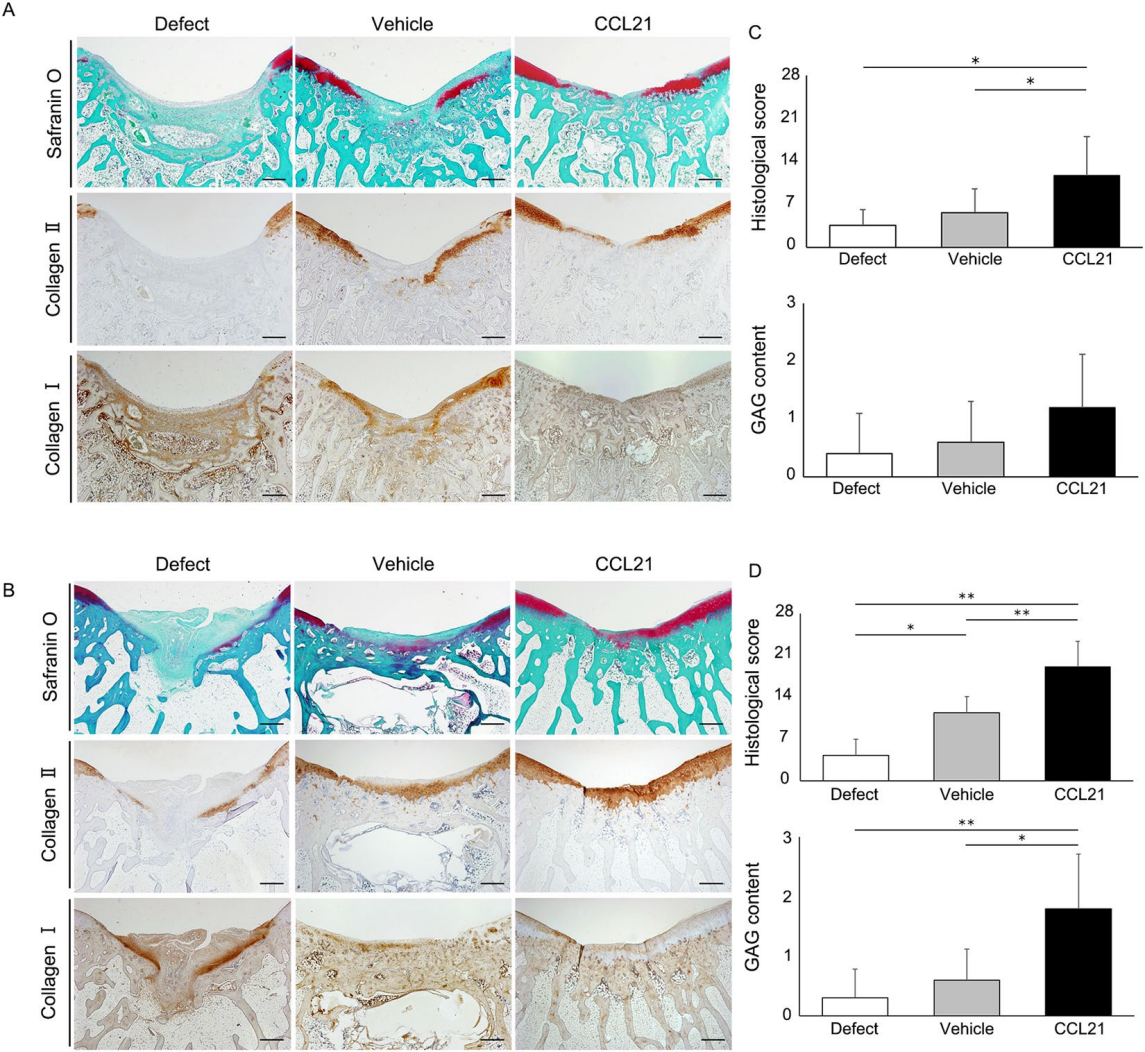
Exogenous CCL21 administration histologically enhances cartilage repair in rabbits. (**A**) Representative images of the patellar groove surface with safranin O staining, immunostaining for type I and type II collagen at 4 weeks postoperatively (n = 10/group). Scale bar: 1 mm. (**B**) Representative images of the patellar groove surface with safranin O staining, immunostaining for type I and type II collagen at 16 weeks postoperatively (n = 10/group). Scale bar: 1 mm. (**C**) Histological scores at 4 weeks postoperatively (n = 10/group). (**D**) Histological scores at 16 weeks postoperatively (n = 10/group). Repair scoring range: 0–28. Histological scores for GAG content (scoring range: 0–3). All values are expressed as mean ± SD. *P < 0.05, **P < 0.01, statistically significant (Steel-Dwass test).

**Figure 7:**
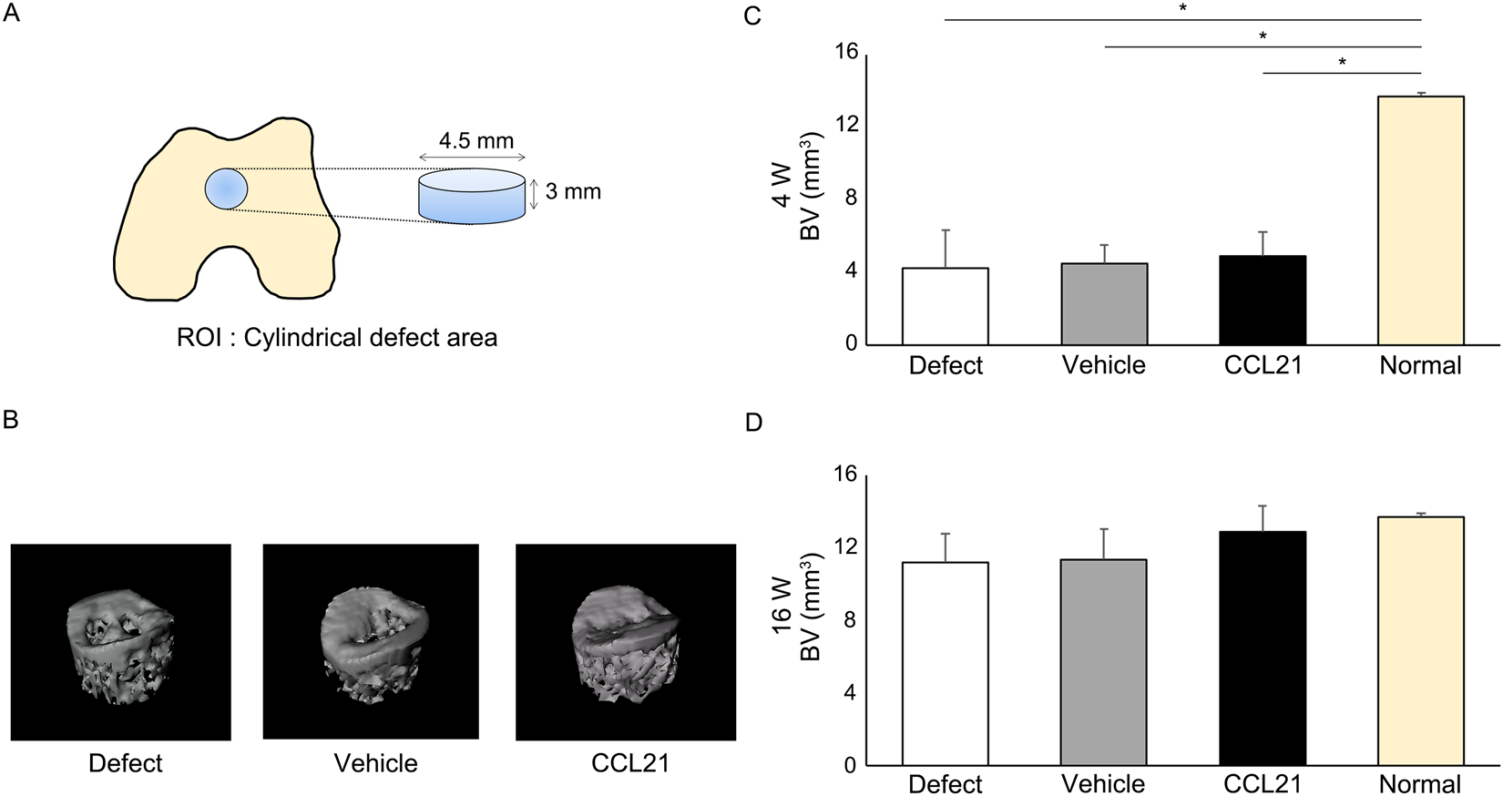
CCL21 does not inhibit subchondral bone repair. (**A**) Evaluation of subchondral bone repair by micro-CT. The cylindrical defect area was assessed as the ROI. (**B**) Representative 3D-CT images at 16 weeks postoperatively from each group (n = 10/group). (**C**,**D**) Repaired subchondral BV was assessed at 4 and 16 weeks postoperatively. All values are expressed as mean ± SD (n = 10/group). *P < 0.05, statistically significant (ANOVA followed by Tukey’s post hoc test).

To confirm the cartilage repair ability of CCL21 *in vivo*, we applied CCL21 embedded into ultrapurified alginate (UPAL^®^) gel to osteochondral defects in adult rabbits. Macroscopic findings at 4 weeks postoperatively indicated that reparative tissue in the defect group was scarcely observed and large depressions remained. In the vehicle group, the osteochondral defects were partially covered with reparative tissue and the surface of the reparative tissue was rough and depressed. In the CCL21 group, the osteochondral defects were mainly covered with reparative tissue except for the centre of the lesion (Fig. 5A). Macroscopic scores in the CCL21 group were significantly higher than the other groups (Fig. 5C). At 16 weeks postoperatively, the osteochondral defects in the defect group were partially covered with reparative tissue and small depressions remained. In the vehicle group, the osteochondral defects were mainly covered with translucent tissue; however, the surfaces of the reparative tissue were moderately rough. In contrast, the osteochondral defects in the CCL21 group were mostly covered with translucent tissue that was sufficiently integrated with the adjacent cartilage (Fig. 5B). The macroscopic scores of the CCL21 group were significantly higher than those of the other groups (Fig. 5D). Histological findings at 4 weeks postoperatively indicated that the osteochondral defects in the defect group were filled with fibrous tissue mainly containing type I collagen. In the vehicle group, the reparative tissue was broadly stained with type I collagen and the bottom of the reparative tissue was slightly stained with safranin O and type II collagen. In the CCL21 group, the osteochondral defects were partially filled with hyaline-like cartilaginous tissue containing glycosaminoglycan (GAG) matrix and type II collagen (Fig. 6A). The histological scores were significantly higher in the CCL21 group compared to the other groups (Fig. 6C). At 16 weeks postoperatively, the osteochondral defects of the defect group were filled with fibrous tissues and exhibited a severely disrupted surface. In the vehicle group, the reparative tissue was mostly fibrous without safranin O staining and the bottom of the reparative tissue was observed to exhibit faint type II collagen staining. Conversely, the reparative tissue in the CCL21 group was mostly hyaline-like cartilage with rich GAG matrix content and type II collagen (Fig. 6B). Furthermore, the histological scores in the CCL21 group were significantly higher than in the other groups (Fig. 6D). Subchondral bone repair of the osteochondral defects in all groups was analysed by micro-computed tomography (micro-CT). At 16 weeks postoperatively, the subchondral bone in all groups was mostly repaired, except for the central region of the defect. There were no significant differences in bone volume among the experimental groups (Fig. 7A–D). Our results revealed that the exogenous administration of CCL21 clearly improved articular cartilage repair in gross morphological and histological evaluations.

## Discussion

Our comprehensive screening, including the expression patterns of cartilage differentiation and long-term cultivation of MSCs, as well as the effect on cell migration, identified the CCL21/CCR7 axis as a candidate chemokine involved in juvenile cartilage repair. We evaluated the spatiotemporal expression pattern of CCL21 during the cartilage repair process. Our study shows that CCL21, CCR7 are transiently expressed in the early stage after cartilage damage in juvenile WT mice that show self-repair of cartilage. It is recognized that chemokines are transiently expressed around injury sites during the tissue repair process^15–17^. Our previous study showed that CXCL12, acting as a chemokine for tissue repair, was transiently expressed in the early phase of tissue injury^11,18^. The expression pattern of CCL21 and CCR7 in juvenile mice closely resembled that of CXCL12 in adults, suggesting that the CCL21/CCR7 axis functions in recruiting progenitor cells to the injury site in juveniles.

To identify functions of CCL21/CCR7 in the self-repair process, an analysis in gene-manipulated mice was required. However, this has previously been difficult because of the lack of a suitable cartilage injury model in gene-manipulated mice. We previously established a cartilage injury model that permits the assessment of the cartilage repair process in gene-manipulated mice^3,19^. Before starting the evaluations, we histologically evaluated the model’s reproducibility and demonstrated that the width of the cartilage injury and the ratio of injury site to articular cartilage were highly consistent between juvenile and adult mice. We have applied this method to CCR7 deficient mice and found that elimination of CCR7 in juvenile mice markedly decreased the capacity for cartilage repair, whereas CCR7^−/−^ adult mice exhibited identical repair with scar-like tissue compared to WT mice. Specifically with respect to juvenile CCR7^−/−^, we observed cartilage destruction involving lower staining of safranin-O and collagen II even though the injury site was of limited size. We first hypothesized that a prominent phenotype possibly occurred in the whole joint due to modulation of protease expression by the CCL21/CCR7 axis^20^. Our results showed that the femoral condyle, a non-injury site, after osteochondral injury showed no significant difference between WT and CCR7^−/−^ mice (Supplementary Fig. S5). Although we also hypothesized that the thin fibrous layer on the articular surfaces are different between these groups, we could not identify any histological differences between them (Supplementary Fig. S4C). Several chemokines have been reported to be related to tissue healing in adults, such as CXCL12, CCL2 and CCL27^11,21,22^; however, there are no previous reports of chemokines related to juvenile tissue healing. It is inferred that the identification of CCR7, which has no effect on adult tissue repair, would have been difficult with conventional analyses. Based on these results, and although the mechanisms of cartilage destruction in juvenile CCR7^−/−^ are still unknown, we concluded that the CCL21/CCR7 axis was a key regulator of the juvenile self-repair mechanism.

In this study, CCL21 was not expressed in the cartilage injury site of adult mice; nevertheless, CCR7 was expressed in adult bone marrow. CCL21 and CCR7 are expressed in chronic articular inflammatory diseases such as osteoarthritis and rheumatoid arthritis^23–25^. Hence, the CCL21/CCR7 axis plays a role in inflammatory responses in adult inflammatory diseases. Sambamurthy *et al*. investigated whether CCR7 deficiency impacted joint degeneration. Histological evaluation showed minimal protection from early cartilage degeneration in CCR7^−/−^ mice at 6 weeks postoperatively; however, there were no significant differences in histological findings at 19 weeks postoperatively^26^. Although it is difficult to make a comparison to this study, it is consistent with our findings that CCR7 contributes to the early phase of the healing process in articular cartilage.

In regards to the cartilage healing process in adults, the CCL21/CCR7 axis may not affect cartilage healing because of the absence of CCL21 expression in injured areas. Based on our results, it was thought that juvenile cartilage repair could be obtained following administration of CCL21 to injured cartilage in adult animals. Thus, we used UPAL^®^ gel to administer CCL21 to adult injury sites. Alginate acid is known to exert a cartilage differentiation effect, and UPAL^®^ gel has reduced endotoxin levels and is suitable for use *in vivo*^11,27–30^. In addition, alginate has been widely used as a scaffold biomolecule in combination with drugs, peptides and proteins. Previous experiments by our group have shown that using UPAL^®^ gel in combination with CXCL12 promoted cartilage repair^11^. In this study, the vehicle group showed faint expression of type I collagen at 4 weeks postoperatively, whereas type I collagen was rarely expressed in the CCL21 group during the early phase of the healing process. These results suggest that the reparative tissue in the control group mainly consisted of fibrous tissue, whereas the reparative tissue in the CCL21 group showed hyaline-like cartilage tissue that contained abundant GAGs. Another report revealed that administration of CCL21 recruited MSCs into skin defects to accelerate wound healing^31^. Based on our findings, we speculated that administration of CCL21 to mature animals would enhance cell migration toward the injury site, with UPAL^®^ gel acting as a bioactive scaffold for chondrogenic differentiation, resulting in scarless cartilage repair. Recent studies have focused on adult tissue repair that employs the self-repair potential of juvenile animals^1,32,33^. Our therapeutic strategy for cartilage injuries is to enhance the natural healing potential using functional biomaterials. We speculate that CCL21 is a key molecule for reproducing scarless healing in adults.

CCL21 accumulated in the injury site during the repair process in juvenile mice; nevertheless, the origin of CCL21 could not be identified. CCL21 is known to exist in lymphoid tissue^24,34^ as well as cartilage^35,36^. We observed that CCL21 was expressed in chondrocytes on the articular surface and growth plate. Additionally, CCL21 was more strongly expressed in chondrocytes of juvenile mice than in those of mature mice. Based on our results, we speculate that CCL21 is possibly secreted by chondrocytes during cartilage injury in juvenile mice. Elimination of CCR7 in mice diminished juvenile cartilage healing, moreover, cartilage destruction was observed beyond the cartilage injury area. The CCL21/CCR7 axis plays a role in T cell migration and establishment of self-tolerance in T cells^37^. Although this study does not examine the immunologic role of the CCL21/CCR7 axis in juveniles, the depletion of CCR7 in juvenile mice caused destruction of articular cartilage, possibly due to an immunologic abnormality. Although MSCs are known to migrate to injury sites following CCL21 binding to CCR7^31,38^, the identity of the CCL21-recruited cells accumulating at the injury site in cartilage is unknown. Since CCL21 significantly enhances migration of MSCs that express CCR7 in bone marrow tissue, we speculate that CCL21 expression in cartilage enhances cartilage repair, possibly due to the recruitment of bone marrow-derived MSCs to the injured site. The application of immunofluorescence tracking assays^39^ has been demonstrated as a method capable of real-time monitoring of gene expression and intracellular biomolecule levels in animal models. However, we were unable to perform a tracking assay because there are currently no established MSC stem cell markers. If optimal MSC markers are available in the future, we will be able to apply a cell tracking assay to resolve the mechanisms.

This strategy may have a great impact on cartilage repair therapy by mimicking self-repair in juvenile individuals. Our findings demonstrate the efficacy of a minimally invasive stem cell-based approach to regenerate articular cartilage.

## Methods

### MSC and chondrocyte culture and chondrogenic differentiation of MSCs

We utilized C57BL/6 mouse MSCs (Cyagen) for the screening experiment and primary MSCs in subsequent experiments. MSCs were harvested from the compact bone of 2-week-old mice, as previously reported^40^. Chondrocytes were cultured in Dulbecco’s modified Eagle’s medium (WAKO) with 10% foetal bovine serum (NICHIREI) and 1% antibiotics (WAKO). Chondrogenic differentiation of MSCs was induced by high-density micromass (2 × 10^5^ cells/10 µl drop)^41^. The concentration of chemokines in the following experiments was 100 ng/ml.

### Quantitative real-time reverse transcriptase-PCR (RT-PCR)

Total RNA was extracted from the samples using the RNeasy Mini kit (Qiagen). For complementary DNA synthesis, 1.0 µg RNA was reverse transcribed using random hexamer primers (Promega) and ImPromII reverse transcriptase (Promega). QPCR was performed using a Thermal Cycler Dice Real Time SystemII (Takara). Signals were detected using SYBR Premix Ex TaqII (Takara) with gene-specific primers (Supplementary Table S1). The relative mRNA expression of each targeted gene was expressed as the Ct value of each gene normalized to the Ct value of GAPDH using the ΔΔCt method^42,43^.

### Comparison of chemokine receptor gene expression in the cartilage differentiation process and long-term cultivation of MSCs

In order to identify the specific chemokines that enhance cartilage repair, we evaluated the expression patterns of 19 chemokine receptors during chondrogenic differentiation and long-term cultivation of MSCs. We evaluated the mRNA expression of chemokine receptors in chondrogenic differentiation. To examine the long-term cultivation of MSCs, we continuously cultured MSCs from passages 9 to 16. We selected the chemokine receptors that exhibited gradually increased mRNA expression with chondrogenic differentiation and decreased mRNA expression after long-term cultivation of MSCs.

### Cell migration assay

We assessed the influence of chemokines on cell migration of MSCs using the CytoSelect^TM^ 96-well cell migration assay (Cell Biolabs), according to the manufacturer’s instructions^44–46^. Feeder tray wells were filled with 150 µl of medium in the presence of seven chemokines (CCL19, CCL21, CCL25, CXCL9, CXCL10, CXCL11, and CXCL12; Peprotech; all chemokines were used at a concentration of 100 ng/ml). Migration assays were run for 8 h at 37 °C in a 5% CO_2_ incubator. The cells were treated with a fluorescent dye (CyQuant^®^) and measured at 480 nm using a SpectraMax M2 Plate Reader (Molecular Devices).

### Cell proliferation assay

To investigate the effect of CCL21 on cell viability, MSCs were cultured in DMEM-HG supplemented with 10% FBS and 1% antibiotics. At 0, 1, and 3 days after cultivation, the number of viable cells at each time point was counted using a Cell Counting Kit-8 (Dojindo Laboratories).

### Experimental Animals

CCR7^−/−^ mice were obtained from The Jackson Laboratory (strain B6; 129P2(C) –Ccr7^tm1Rfor/J^; stock number 006621). C57BL/6 mice were purchased from Japan SLC, Inc. and used as wild type (WT) mice. Japanese white rabbits were purchased from Japan SLC, Inc. All animal experiments were performed on females.

### Generation of cartilage injury in mice and rabbits

Articular cartilage full-thickness injuries were generated in juvenile (3-week-old) and adult (8-week-old) female mice as previously reported (n = 10/group)^3^. Cartilage injuries were generated using a 27G needle. Longitudinal full-thickness injuries along the femoral axis were made in the patellar groove using the end of the needle. Osteochondral defects were generated in 16-week-old female Japanese white rabbits, as previously reported^11^. A full-thickness osteochondral defect 4.5 mm in diameter and 3 mm in depth was created in the patellar groove of each knee. An *in situ* forming material based on UPAL^®^ gel with a molecular weight of 1700 kDa (Sea Matrix®; Mochida Pharmaceutical Co., Ltd.) was used in this experiment^28^. The articular cartilage defects were divided into three groups (n = 10/group) as follows: defect, no treatment; vehicle, UPAL^®^ gel containing 10 µg/mL bovine serum albumin; CCL21, UPAL^®^ gel containing 10 µg/mL CCL21 (Peprotech).

### Tissue processing and histology

The animals were euthanized for further investigation at each time point. To observe the skeletal system, whole skeletons of WT and CCR7^−/−^ littermates were stained with alcian blue and alizarin red^47^. The knee joints were fixed with 10% formalin (WAKO), and then decalcified with ethylenediaminetetraacetic acid, embedded in paraffin, and sectioned at 5-µm thickness. To observe the repair of cartilage injury, histological sections were made as previously reported^3,27,48^. In the rabbit experiment, sections were obtained from the centre portion of each defect. The sections at each time point were used for histomorphometry and scoring. All slides were analysed using an Olympus DP72 camera and DP2-BSW software (Olympus).

### Macroscopic and histological evaluations of cartilage repair

In the mouse experiment, the animals were euthanized for further investigation at each time point. The histological score for cartilage repair of individual sections was evaluated as previously reported^49,50^. The scoring was performed independently by two blinded observers. In the rabbit experiment, the animals were euthanized for further investigation at 4 and 16 weeks after the operation. The knee samples were obtained and photographed with a digital camera for macroscopic evaluation. The specimens were subsequently prepared for histological analyses. The macroscopic and histological findings were evaluated as previously reported^51^. For quantitative evaluation of GAG content, the findings of safranin O staining were scored using Mohan’s scoring system^52^. Scoring was performed independently by two blinded observers.

### Immunohistochemical analysis

In the mouse experiment, sections were incubated with the primary antibody anti-type II collagen (Daiichi Fine Chemical). In the rabbit experiment, sections were incubated with the primary antibodies anti-type I (abcam) and anti-type II collagen (Fuji Pharm.). The samples were incubated with a biotinylated secondary antibody and visualized by the precipitation of diaminobenzidine (DAKO). For CCL21 and CCR7 evaluation, sections were incubated with the primary antibodies anti-CCL21 (R&D), anti-CCR7 (Abcam), and staining was visualized with Alexa Fluor 568 (Invitrogen), FITC Conjugated AffiniPure (Jackson Immuno Research). Nuclei were stained with DAPI (Invitrogen).

### Micro-CT evaluation of subchondral bone repair

The harvested tissues were evaluated with a micro-CT scanner (R mCT; Rigaku) at a resolution of 20 µm/pixel. The imaging conditions of micro-CT were 80 kV and 100 µA. The images were processed with Image J software (National Institutes of Health, Bethesda, MD), and quantitative 3D bone analyses were performed using the Bone J plugin for Image J^53^. Regions of interest (ROI) were manually set at the original cylindrical defect area (4.5 mm in diameter and 3.0 mm in depth). The volume of mineralized bone, defined as the repaired bone volume (BV) in the ROI, was calculated.

### Statistical analysis

All statistical analyses of the data were performed using the statistical software JMP Pro 11.0. All data are presented as means ± standard deviation. Significant differences among three or more groups were assessed by one-way ANOVA followed by Tukey’s post hoc tests. Statistical comparison of data between the two groups of samples was performed with an unpaired t-test. Mean histological scores were statically compared using non-parametric Steel-Dwass tests between pairs of groups. Significance was accepted with a P value < 0.05.

### Study approval

All experiments were performed according to a protocol approved by the Institutional Animal Care and Use Committee of the Hokkaido University Graduate School of Medicine.

## Supplementary information

Dataset 1
