# Supplementary material for: CCL21/CCR7 axis regulating juvenile cartilage repair can enhance cartilage healing in adults
Source: Sci Rep. 2019 Mar 26;9:5165. doi: 10.1038/s41598-019-41621-3 (PMC6435673; doi:10.1038/s41598-019-41621-3)
Supplement: Supplementary file 1 — Dataset 1 [file 41598_2019_41621_MOESM1_ESM.docx]

**CCL21/CCR7 axis regulating juvenile cartilage repair can enhance cartilage healing in adults**

Zenta Joutoku1, Tomohiro Onodera1, 2, Masatake Matsuoka1, Kentaro Homan1, Daisuke Momma1, Rikiya Baba1, Kazutoshi Hontani1, Masanari Hamasaki1, Shinji Matsubara1, Ryosuke Hishimura1, and Norimasa Iwasaki1, 2

^1^ Department of Orthopaedic Surgery, Faculty of Medicine and Graduate School of Medicine, Hokkaido University, Sapporo, Japan

^2^ Global Station for Soft Matter, Global Institution for Collaborative Research and Education (GSS, GI-CoRE), Hokkaido University, Sapporo, Japan

**Supplementary figure legends**

**Supplementary Figure S1. Chondrogenic differentiation from MSCs.**

qRT-PCR analysis of the chondrogenic differentiation process **(A-C)**

**(A)** *Sox9* mRNA, **(B)** *Col2a1* mRNA, **(C)** *Aggrecan* mRNA (n = 3 triplicates per group). All values are expressed as mean±SD.

**Supplementary Figure S2. Quantitative evaluation of CCL21 in articular cartilage of WT mice.**

**(A)** Left panels: Representative images for immunohistochemistry of CCL21 (red) in juvenile WT mice. Scale bar: 200 µm. Right panels: Representative images for immunohistochemistry of CCL21 (red) in adult WT mice. Scale bar: 200 µm. **(B)** CCL21 positive cell ratios determined by counts of CCL21 positive cells and DAPI within each of three fields (1 unit area: 1 mm^2^). All values are expressed as mean±SD. *P < 0.05, statistically significant (t test). **(D)** CCL21 positive area determined by calculating the CCL21 positive area within each of three fields (1 unit area: 1 mm^2^). All values are expressed as mean±SD. *P < 0.05, statistically significant (t test).

**Supplementary Figure S3. CCL21 positive cell numbers at injury sites in WT mice.**

**(A, B)** Representative images of injury sites in juvenile and adult WT mice with safranin O staining, immunohistochemical staining for CCL21 at 3 days postoperatively (A: juvenile, B: adult) (n = 4/group). Scale bar: 50 µm. **(C)** CCL21 positive cell numbers determined by counts of CCL21 positive cells at the injury site. All values are expressed as mean±SD. *P < 0.05, statistically significant (t test) (n = 4/group).

**Supplementary Figure S4. Normal growth and development of young CCR7^-/-^ mice.**

**(A)** Double staining of the whole skeleton in newborn WT and CCR7^-/-^ mice with Alcian blue and alizarin red (n =2). Scale bar: 1 cm. **(B)** Growth curves, determined by body weight, in female WT and CCR7^-/-^ mice. Body weights were not significantly different between the genotypes (n =10/group) (t test). All values are expressed as mean±SD. **(C)** Histological findings in knee joints from 4-week-old mice. Safranin O staining and immunostaining for type II and type Ⅰ collagen in articular cartilage were performed in each mouse genotype (n = 10/group). Scale bar: 100 µm **(D)** Articular cartilage thickness did not differ significantly between genotypes (n =10/group). All values are expressed as mean±SD (t test). **(E)** Histological findings in knee joints from 4-week-old mice. Toluidine blue (TB) staining and immunostaining for type II collagen in the tibial growth plate were performed in each mouse genotype (n = 10/group). Scale bar: 100 µm **(F)** Growth plate width did not differ significantly between the genotypes (n =10/group). All values are expressed as mean±SD (t test).

**Supplementary Figure S5. Evaluation of femoral condyle after osteochondral injury.**

**(A, B)** Representative images of the femoral condyle in juvenile mice with safranin O staining and H&E staining at 8 weeks postoperatively (A: medial, B: lateral) (n = 5/group). Scale bar: 100 µm. **(C, D)** Mankin scores at 8 weeks postoperatively (n = 5/group). Mankin scoring range: 0-14. All values are expressed as mean±SD.

**Supplementary Figure S6. Association between elimination of CCR7 and chondrocyte behaviour *in vitro*.**

**(A)** The effect of CCL21 (100 ng/ml) on cell migration (n = 10/group). All values are expressed as mean±SD. *P < 0.05, statistically significant (ANOVA followed by Tukey’s post hoc test).

**(B)** The effect of CCL21 (100 ng/ml) on cell proliferation (n = 10/group at each time point). All values are expressed as mean±SD.

**Supplementary Table S1. Primers used for real-time RT-PCR**

**Supplementary Figure S1**


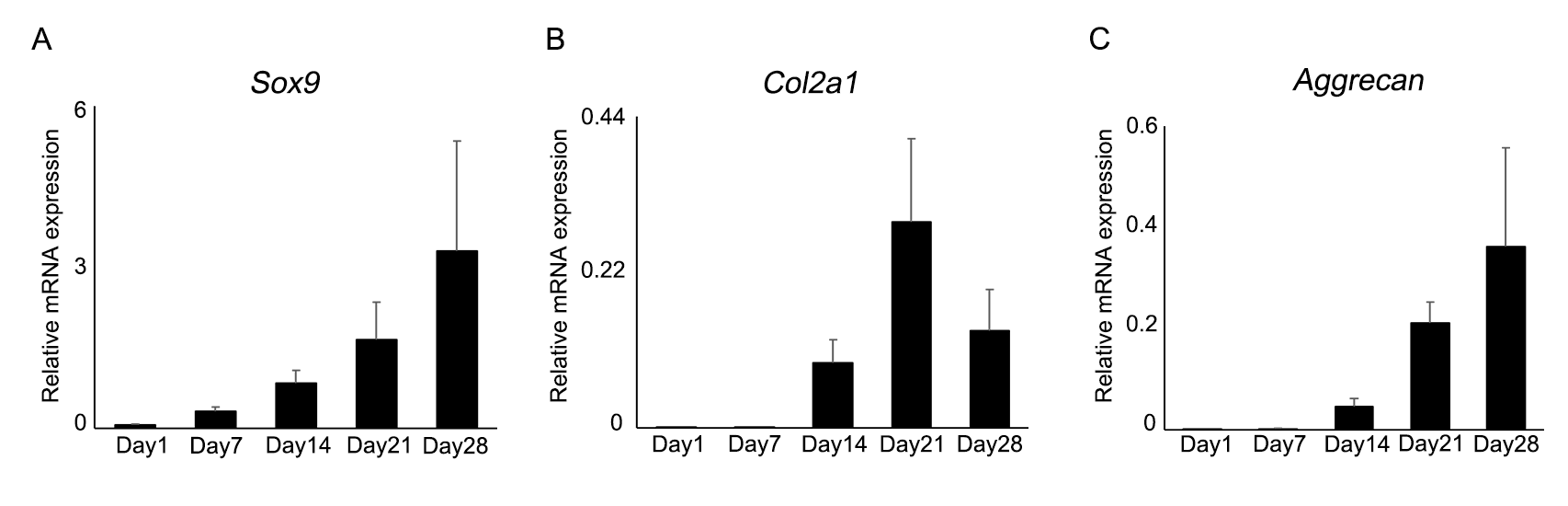


**Supplementary Figure S2**


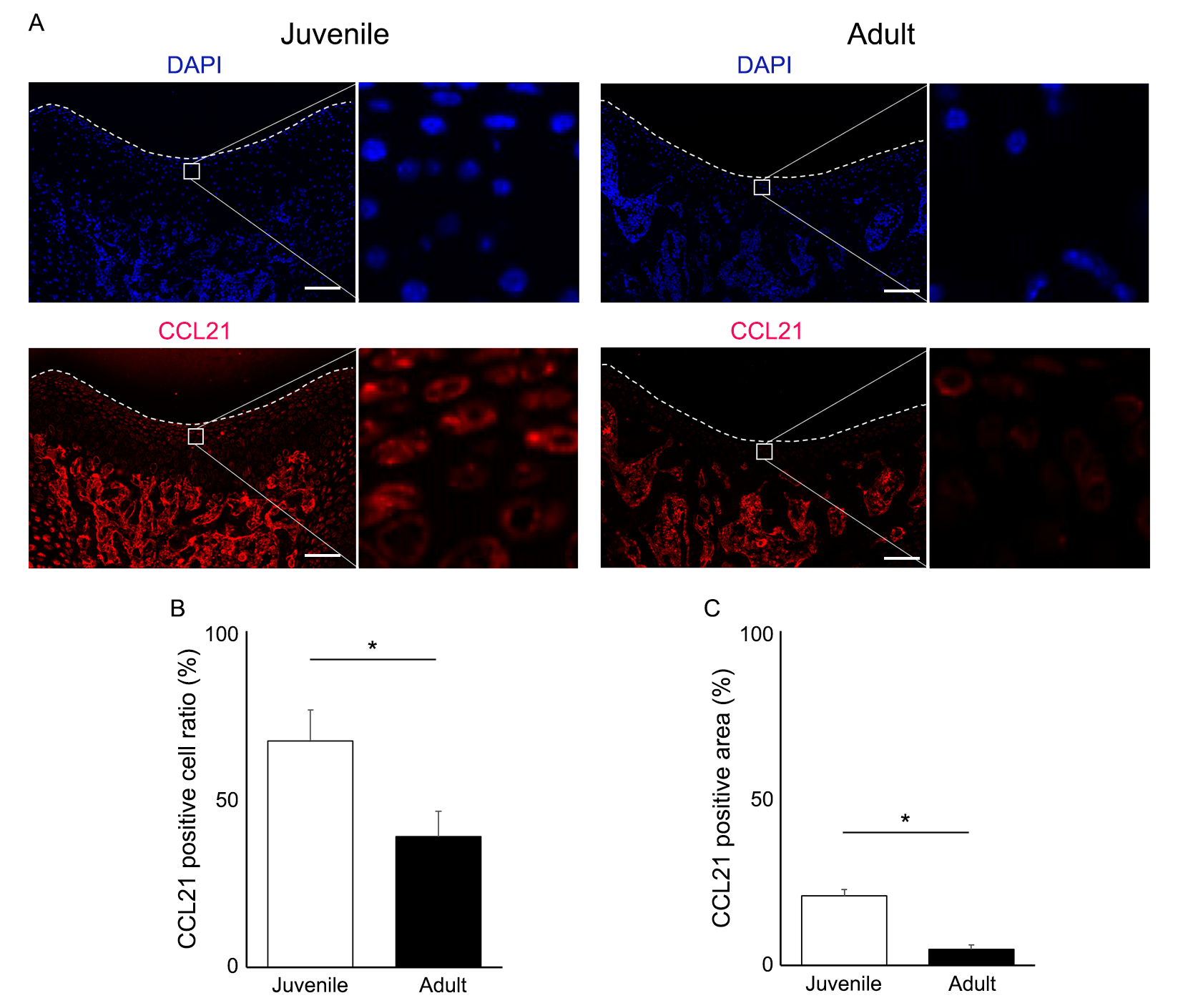


**Supplementary Figure S3**


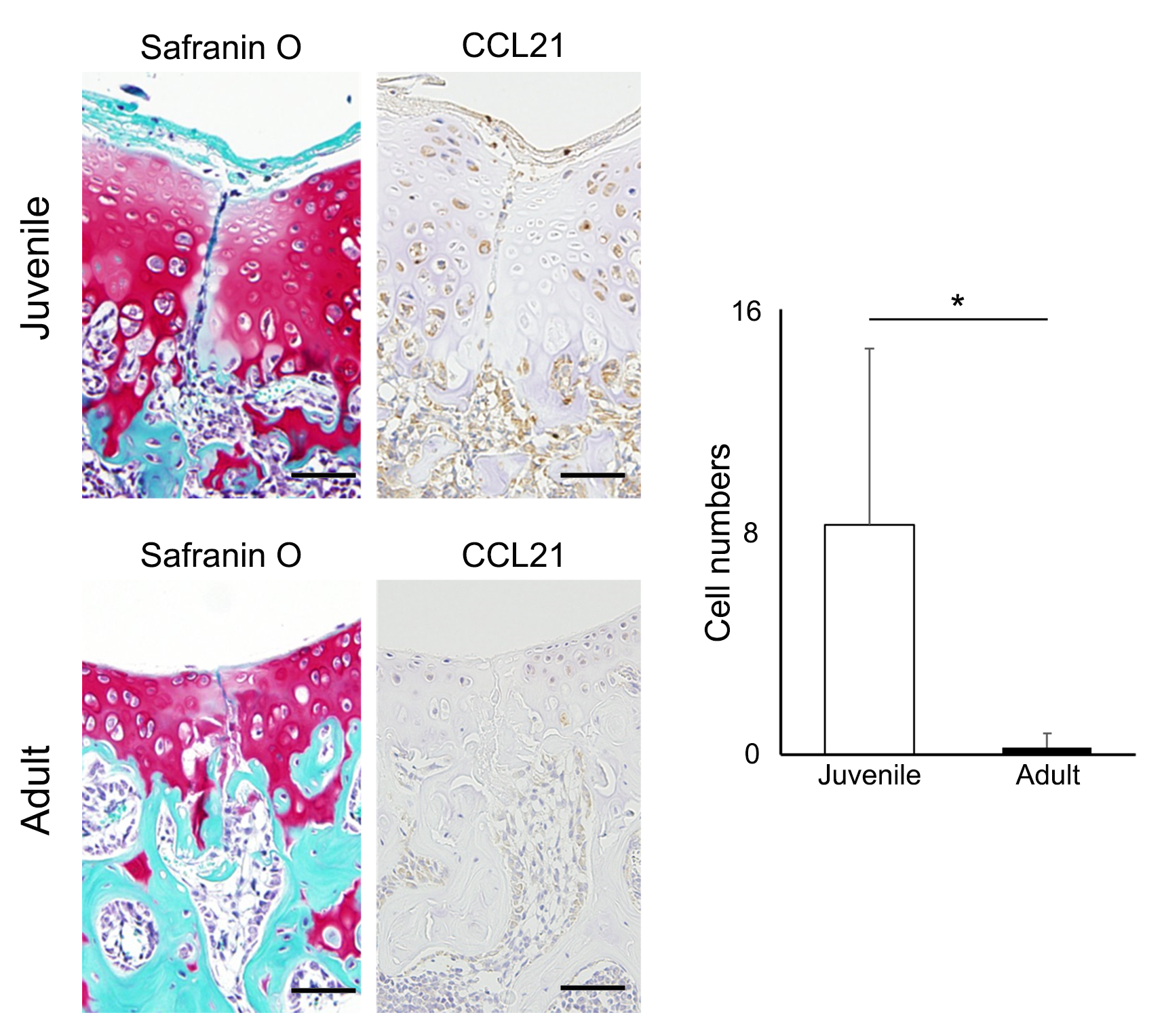


**Supplementary Figure S4**


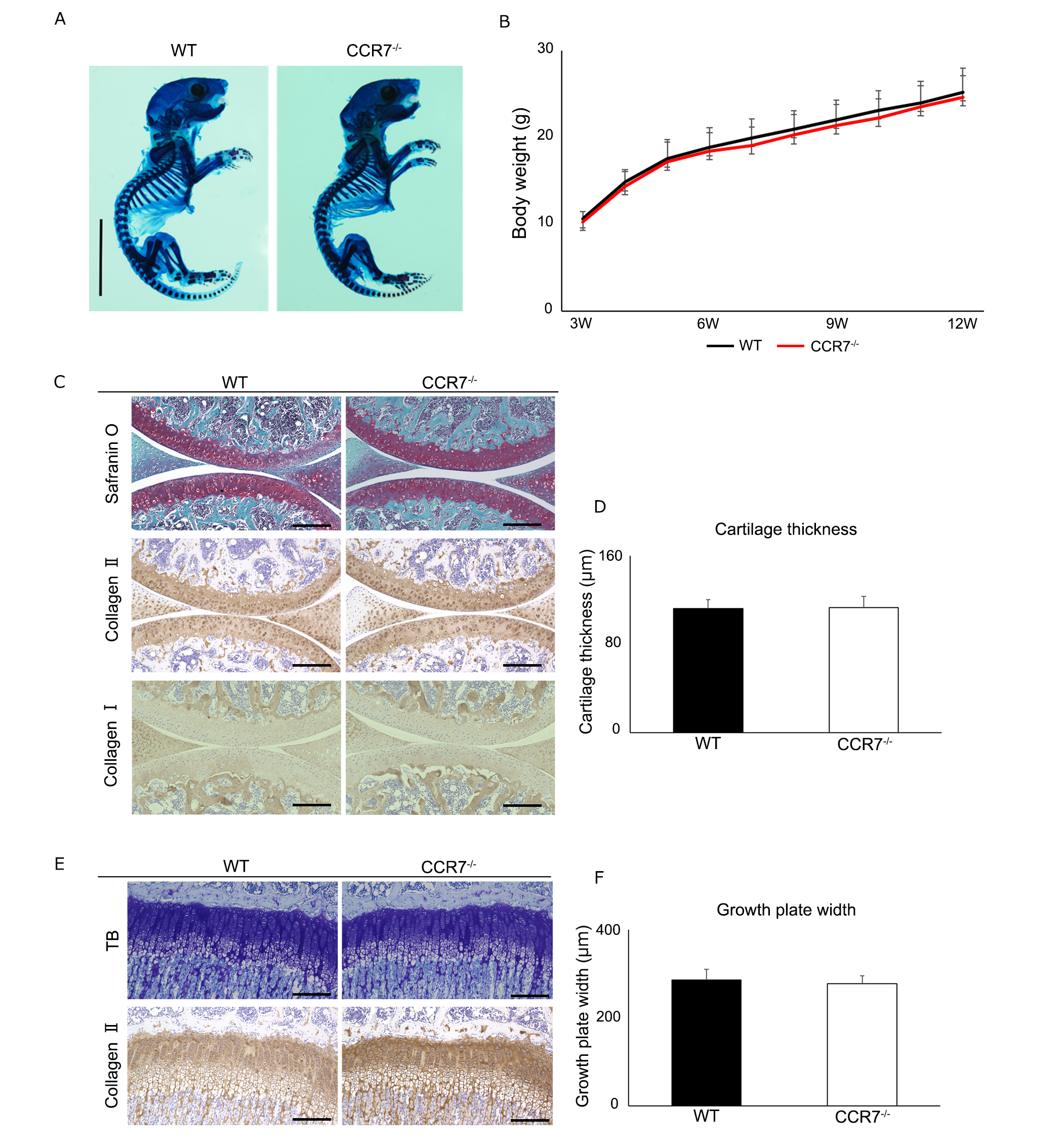


**Supplementary Figure S5**

**
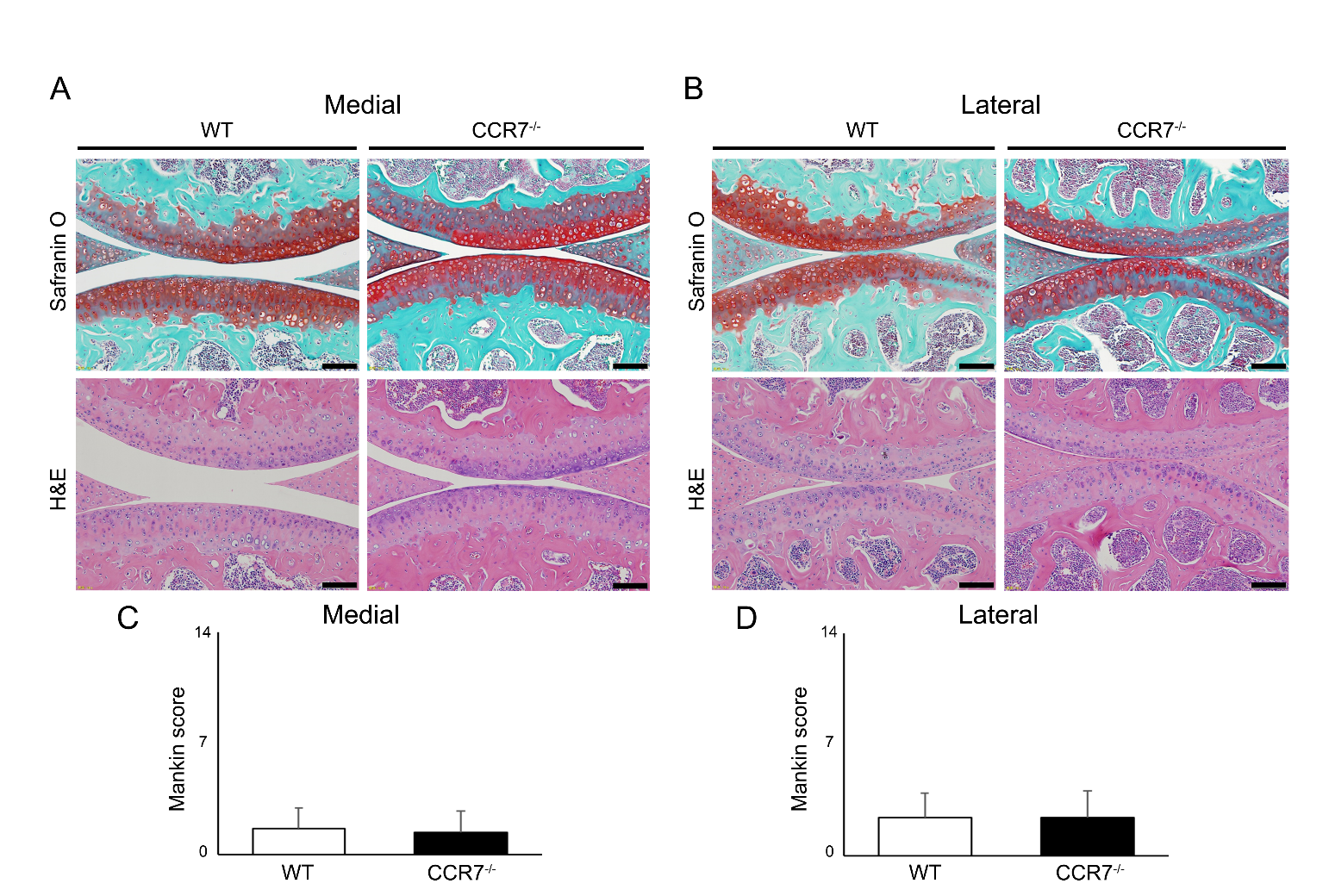
**

**Supplementary Figure S6**


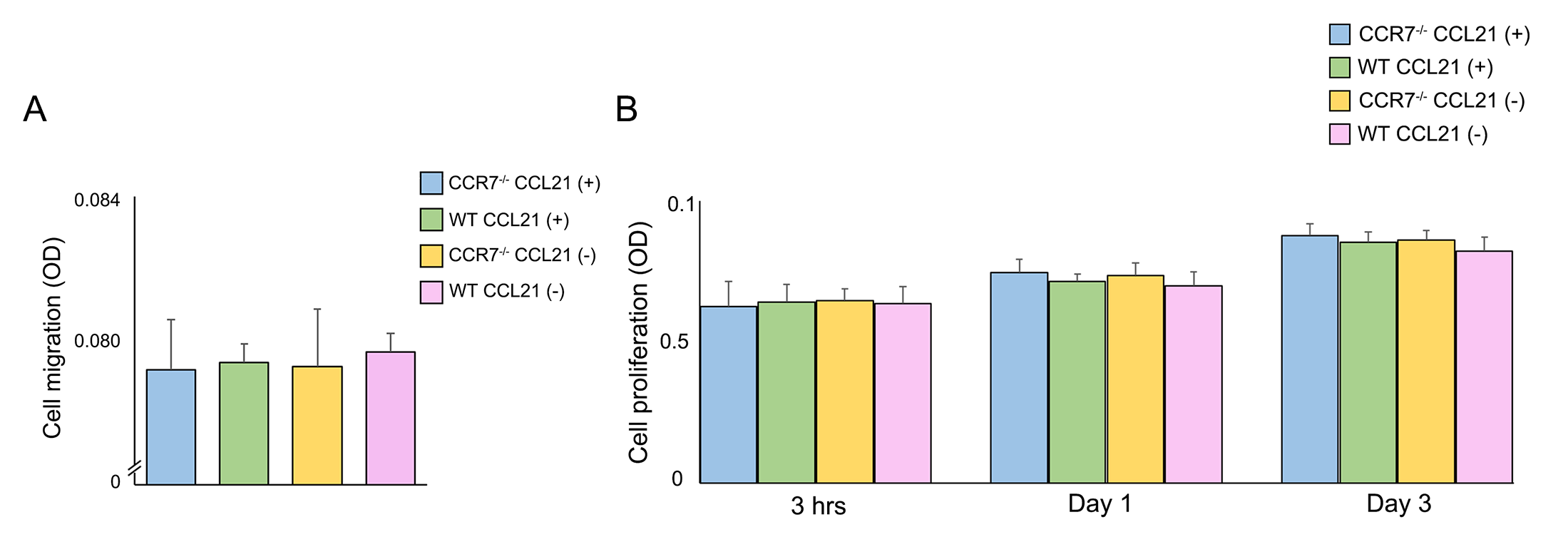


**Supplementary Table S1**

|  | Forward | Reverse |
| --- | --- | --- |
| CCR1 | 5’ AGGCCCAGTGGGAGTTCAC 3’ | 5’ TCCACTGCTTCAGGCTCTTGT 3’ |
| CCR2 | 5’ TCATCCACGGCATACTATCAACA 3’ | 5’ GTGGCCCCTTCATCAAGCT 3’ |
| CCR3 | 5’ GCTTTGAGACCACACCCTATGAA 3’ | 5’ GACCCCAGCTCTTTGATTCTGA 3’ |
| CCR4 | 5’ AGAAAATTCATGCGAGGGAGAA 3’ | 5’ TGTCACTCAAGGGTCGTGTCA 3’ |
| CCR5 | 5’ AGGCCATGCAGGCAACAG 3’ | 5’ TCTCTCCAACAAAGGCATAGATGA 3’ |
| CCR6 | 5’ GAACTGCCCACTTCCCTTTCT 3’ | 5’ CAGGCTGGCGTGGTTCTCT 3’ |
| CCR7 | 5’ GCTCCAGGCACGCAACTTT 3’ | 5’ GACTACCACCACGGCAATGA 3’ |
| CCR8 | 5’ TGACCGACTACTACCCTGATTTCTT 3’ | 5’ GCTGCCCCTGAGGAGGAA 3’ |
| CCR9 | 5’ TCTCAGTTCCCCTACAACTCCATT 3’ | 5’ CAGTTGGAGATGAACATGGCATA 3’ |
| CCR10 | 5’ TGCTCCTACTGAGACCCA 3’ | 5’ CCCTGGGATTGTTTCTTT 3’ |
| CXCR1 | 5’ CCCTCTTTAAGGCCCACATG 3’ | 5’ AAGGACGACAGCGAAGATGAC 3’ |
| CXCR2 | 5’ ACCCTCTTTAAGGCCCACATG 3’ | 5’ CAAGGACGACAGCGAAGATG 3’ |
| CXCR3 | 5’ CAGCCTGAACTTTGACAGAACCT 3’ | 5’ GCAGCCCCAGCAAGAAGA 3’ |
| CXCR4 | 5’ TCAGCCTGGACCGGTACCT 3’ | 5’ GCAGTTTCCTTGGCCTTTGA 3’ |
| CXCR5 | 5’ GCCTGCTCGTGGCCTGTA 3’ | 5’ CGGCGGTAGGCGTGAAC 3’ |
| CXCR6 | 5’ TACGATGGGCACTACGAGGGAG 3’ | 5’ GCAAAGAAACCAACAGGGAGACCAC 3’ |
| CXCR7 | 5’ GAGGTCACTTGGTCGCTCTC 3’ | 5’ GTGTCCACCACAATGCAGTC 3’ |
| XCR1 | 5’ GCCTTCTCTCATTGCTGTTTCA 3’ | 5’ TTTAGGTGTCTGCGGAACTTGA 3’ |
| CX3CR1 | 5’ TCAGCATCGACCGGTACCTT 3’ | 5’ CTGCACTGTCCGGTTGTTCA 3’ |
| Sox9 | 5’ AGACAGCCCCCTATCGACTT 3’ | 5’ CGGCAGGTACTGGTCAAACT 3’ |
| Col2a1 | 5’ GGCAATAGCAGGTTCACGTACA 3’ | 5’ CGATAACAGTCTTGCCCCACTT 3’ |
| Aggrecan | 5’ TCGAGGACAGCGAGGCC 3’ | 5’ TCGAGGGTGTAGCGTGTAGAGA 3’ |
